# Numerical simulation of bubble rising behavior in a tannin-based foaming precursor resin

**DOI:** 10.1016/j.heliyon.2024.e40292

**Published:** 2024-11-09

**Authors:** Lan Huang, Haizhu Wu, Wenbin Yuan, Hisham Essawy, Guanben Du, Xiaojian Zhou, Xinyi Chen

**Affiliations:** a Yunnan Provincial Key Laboratory of Wood Adhesives and Glued Products, Southwest Forestry University, Kunming, 650224, China; b Key Laboratory for Forest Resources Conservation and Utilization in the Southwest Mountains of China, Southwest Forestry University, Kunming, 650224, China; c Department of Polymers and Pigments, National Research Centre, Cairo, 12622, Egypt

**Keywords:** Bubble behavior, Tannin-based foaming precursor resin, Two–phase flow, VOF model, Numerical simulation

## Abstract

A two-dimensional volume of fluid (VOF) model was developed to simulate the deformation of the bubble, the end speed of bubble rise, the distance of bubble rise and the movement trajectory in different initial conditions of tannin-based foaming precursor resin. In this study, bubble rising and coalescence characteristics are connected with parameters of the resin, especially viscosity, surface tension, the initial radius and location of the bubble also matter. The result shows that rising velocity of the bubble decreased as the viscosity increased, and at the same time, the flow rate of the bubble was lower. In addition, with the increase in surface tension, the ability of the bubble to change shape was impeded, whereas when the bubble radius increased, the rising velocity of the bubble was faster. The floating behavior of parallel double bubbles and coaxial double bubbles in resin was simulated. It was found that the distance had important effects on their coalescence behavior. For parallel doubles, the motion process is symmetric, because of their vortices, the bubbles will move away from each other, and their motion properties depend on the relative position of the bubbles which is crucial for the bubble merger. For coaxial double bubbles, the closer the distance between the two bubbles is, the faster the fusion speed will be. When the distance is fixed, the larger the radius is, the shorter the fusion time will be, which proves that there are differences in the growth rate of bubbles during the foaming process.

## Highlights

• CFD technique was utilized in tannin based foaming precursor resin.
• A way to reduce bubble escape was suggested in this work.
• Double bubbles motion characteristic was obtained by CFD method.
• This work provides theoretical guidance for obtaining good performance tannin-based foam.

## Graphical abstract

**Figure:**
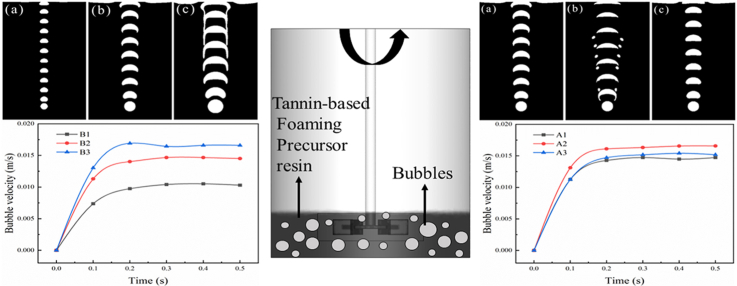

## 1 Introduction

Tannin foams have been widely investigated due to their outstanding performance, such as lightweight, inflaming retarding, and thermal-insulation and other excellent properties that can be applied to the building packaging industry [1]. The foam prepared by Pizzi et al. [2] has good mechanical properties, but the foam microstructure is not uniform. Tondi et al. [3] used ether as a foaming agent to quickly prepare tannin foam materials, successfully solving the problem of difficult foaming. However, the prepared foam materials also had the problem of uneven cell distribution in structure. During the preparation of tannin foam materials, a large number of bubble nuclei will appear, and the distribution of these bubble nuclei can determine whether the cellular structure of the prepared foam is uniform [4]. The preparation of tannin foams often includes raw materials such as tannin, formaldehyde, furfuryl alcohol, foaming agent and acid catalyst [5]. Tannin foam has a broad development prospect due to its good performance and small burden on the environment. However, there are many factors affecting the performance of tannin foam materials, among which the pore structure of foam has a significant impact on its mechanical properties, while the pore structure of tannin foam materials is closely related to its foaming process, and the foaming process of tannin foam materials belongs to the field of fluid mechanics. Therefore, the method of computational fluid dynamics is considered to study the foaming process of tannin foams. These properties are normally related to the foam cell structure, which is strongly influenced by the foaming behavior in each method, especially in the case of foaming induced by mechanical stirring. Compared to the traditional free foaming process by blowing agent, mechanical stirring induced foaming strategy was considered sustainable because the blowing agent was removed. Additionally, the foaming process was easily controlled by adjusting the formulation and mechanical stirring parameters, including the type of stirrer, the concentration of tannin-based foaming precursor resin, and the speed of stirring, etc.

Tannin-based foam preparation by mechanical stirring, by expressed, is a motor behavior journey for air bubbles in tannin precursor. Plenty of air was captured by tannin resin precursor due to paddle agitate intensely, forming a mixture of air bubbles and tannin resin. Therefore, it was generally involved in air bubble evolution and cell structure fixing process which was related to gas–liquid two–phase flows, high temperature, and laminar flow [[6], [7], [8], [9], [10], [11]]. However, these two processes occur almost simultaneously during the preparation, resulting in a huge block for obtaining specific experimental data required for a comprehensive understanding of the foaming process.

Numerical simulation was normally utilized to study the bubble movement characteristics for a better understanding of the influence of tannin-based foaming precursor resin and bubble properties [[12], [13], [14], [15]]. The gas–liquid two–phase system, in the few past decades, has been considered a classical foam materials precursor resin model for analyzing the bobble movement behavior in the foaming precursor, by using computational fluid dynamics (CFD) technology. Generally, the two–phase flow model, including the homogeneous flow model, separated flow model, variable density model, drift flow model, and two–fluid model, have been widely studied by further investigating the changes in properties such as the distribution of bubble size, gas holdup, and velocity fluctuation [[16], [17], [18]]. By contrast, the numerical simulation showed advancement that could not be achieved for the offline experiment as the bubble trajectory, rising velocity and shape morphology could be visualized effortlessly [[19], [20], [21], [22], [23], [24], [25], [26]]. Ali F. Abu-Bakr proposed the theoretical and mathematical approaches of lipid-coated multi-microbubbles in viscoelastic tissues during the growth process of microscopic bubbles. Investigate the growth of lipid-coated multi-microbubbles under the effect of constant and variable surface tension, and some physical parameters are affected as a result of the bubble-bubble interaction [27]. At the same time, the complex phenomena of the mechanical stirring foaming process for tannin foam can be simplified as the behavior of bubbles in tannin-based foaming precursor resin.

It is essential to concentrate on the interface between the bubble and tannin-based foaming precursor resin when studying the bubble behavior because the two phases cannot fuse, and it is difficult to measure the geometrical shape of the fluid interface. Hence the experimental method to obtain the interface is a hard problem, however, with the development of computational fluid dynamics, there have been plenty of methods to track the gas–liquid interface, such as the Particles in Fluid Method first studied by Harlow and Welch [28] and the Level Set Method put out by Osher and Sethian [29]. As well-known numerical methods, the VOF model can simply and accurately track the interface [30]. There have been papers by Hirt and Nichols [31] in which the VOF method was first proposed with SOLA–VOF code to address complicated free boundary configurations and track free fluid surfaces. It is easy to understand the VOF method that the large density of the flow phase is set to be the primary phase and an interrupted scalar function is led into the whole flow field defined F, which is a real number and the value is from zero to one. When the value is one, the unit of the mesh part contains just the primary flow phase, and if the value is zero, the unit of the mesh part is just the second flow phase. When the value is in the range of zero and one, the unit of the mesh part has both flow phases; at the same time, the interface is located in the mesh part. To obtain the flow field distribution, the transportation equation of the volume fraction is calculated. It is very suitable in engineering to use the VOF method due to its faster calculation speed and lower computational resources. In recent studies with the VOF model to simulate gas–liquid two-phase flow problems, Seong-Su Jeon [32] made use of the VOF method to simulate the behavior of condensing bubbles in subcooled boiling flow to analyze the effects of condensation on bubble behavior. It was found that the simulation results showed good agreement with the experiment performed. The condensation increases the bubble velocity. As the condensation rate increases, the bubble is reduced but travels a longer distance, and the bubble condensation accelerates the lateral migration of the bubble so that the VOF model can be applied in predicting the bubble shape, terminal velocity and moving trajectory. Additionally, GUILI GAO [33] presented a two-dimensional VOF model to simulate the effect of rotation speed and ultrasonic parameters on behavior under a compound field, and the experimental results, including bubble shape and trajectory, were the same as the experimental observations. Moreover, Yong Li [34] analyzed a single bubble rise behavior at pressures of 0.1 and 19.4 MPa in a bubble column, and volume tracking based on the VOF method was used to describe the interface between gas and liquid, which was satisfactory compared with the experimental results. However, the simulation is limited because it only compares several cases and the empirical parameters are given under certain conditions. The problem of bubble formation at submerged orifices under constant inflow conditions was studied by D. Gerlach [35], the formation process was simulated in a combined VOF and level–set method, and the numerical results were extensively validated with literature. Furthermore, ZHAO Xi–zeng [36] simulated extreme generation by using the VOF method in a two–dimensional model, and the results were compared with the theoretical results, concluding that the VOF model can describe practical purposes. The structure multiblock and overset grids were studied with the VOF method and showed that the method can reduce the spurious velocity [37]. The VOF method in CFD can be thought to reconstruct the interface of gas–liquid two–phase with movement of the bubble, which is more flexible and efficient in tracking the interface.

Herein, to understand the bubble rising and coalescence characteristics, the two–phase flow was modeled using the VOF model in a laminar flow, and the model region was validated with literature before calculating and comparing with the theoretical results. According to the model computed by using the volume of fluid model, the bubble behavior, including bubble deformation, terminal velocity, rising distance and moving trajectory, was investigated under various conditions.

## 2 Mathematical model

Tannin-based foam prepared by mechanical stirring foaming approach was considered to understand the air bubble rising behavior in the tannin-based foaming precursor resin under intensive stirring. As described in the former, the foaming system is a mixture of air bubbles and tannin resin. Any ultimate formative foam cell structures are based on the air bubbles which can be a single original or a fusion of two adjacent bubbles and so on. It is a complex process for calculation without basic research, so the work was divided into two parts, the bubble rising behavior in still and mechanically stirred tannin-based foaming precursor resin, respectively. As it is known to all, the volume of fluid model can precisely track the gas-liquid interface, so it can be used for investigating the trajectory of the bubble by tracking the interface of tannin-based foaming precursor resin and bubbles. Therefore, the volume of fluid method was used to simulate rising behavior of the bubble in tannin-based foaming precursor resin.

In this work, the VOF model in the commercial CFD code FLUENT 2022R1 is used to simulate the gas–liquid two–phase flow in still tannin-based foaming precursor resin to determine the rising behavior of the bubble. The post processing is dealt in TECPLOT360. The coupling of pressure and velocity is implemented by SIMPLE algorithm. The momentum is second order upwind and the discrete format of volume fraction is first order implicit. The time step was at 0.001 to ensure the convergence of the solution process. The calculation was deemed to be complete when the residuals of all variables were less than 0.001. The floating speed is slower for the bubble in tannin-based foaming precursor resin with high viscosity, so the flow is assumed to be laminar. In addition, gas–liquid two–phase fluids are considered incompressible fluids. Because more attention is given to the physical properties of tannin-based foaming precursor resin in the process of floating, the change in the energy is not considered. Therefore, the simulation can be considered a transient flow process, which is controlled by the continuity equation and the momentum conservation equation, and the two–dimensional forms are as follows:

**Governing Equations**

(1) $$\begin{array}{c} \frac{\partial \rho }{\partial t}+\rho \left(\frac{\partial v_{x}}{\partial x}+\frac{\partial v_{y}}{\partial y}\right)=0 \end{array}$$

**Momentum conservation equation**

(2) $$\begin{array}{c} \rho \left(\frac{\partial v_{x}}{\partial t}+v_{x}\frac{\partial v_{x}}{\partial x}+v_{y}\frac{\partial v_{x}}{\partial y}\right)=\rho g_{x}-\frac{\partial p}{\partial x}+\eta \nabla^{2}v_{x} \end{array}$$

(3) $$\begin{array}{c} \rho \left(\frac{\partial v_{y}}{\partial t}+v_{x}\frac{\partial v_{y}}{\partial x}+v_{y}\frac{\partial v_{y}}{\partial y}\right)=\rho g_{y}-\frac{\partial p}{\partial y}+\eta \nabla^{2}v_{y} \end{array}$$

where $v_{x}$, $v_{y}$ is the velocity of orientation of x, y, $m\cdot s^{-1}$; p is the pressure, $N\cdot m^{-2}$; η is the motion viscosity, Pa⋅s; g is gravity, $m\cdot s^{-2}$; ρ is the density of the fluid, $kg\cdot m^{-3}$.

### 2.1 Continuum surface force model (CSF)

The surface tension is taken into account in the flow of the bubble, so the CSF model [38] is used to add the interfacial tension term to the equation of conservation of momentum.

(4) $$\begin{array}{c} n=\nabla \alpha_{q} \end{array}$$

(5) $$\begin{array}{c} k=\nabla \cdot n \end{array}$$

(6) $$\begin{array}{c} \hat{n}=\frac{n}{\left|n\right|} \end{array}$$

Where n is the surface normal vector, $\alpha_{q}$ is the gradient, and the volume fraction of phase q, k is the curvature. Surface tension is defined by the pressure jump of the surface. The force on the surface can be expressed as a body force using the divergence theorem, which is the source item and is added to the momentum equation:

(7) $$\begin{array}{c} F=\sigma_{ij}\frac{\rho k_{i}\nabla \alpha_{i}}{\frac{1}{2}\left(\rho_{i}+\rho_{j}\right)} \end{array}$$

where ρ is the density average of the volume, $kg\cdot m^{-3}$; from above, the surface tension source item is proportional to the average density within the unit. The interface between the two gas–liquid phases was tracked to obtain the trajectory of the bubble in the tannin liquid. The VOF model, in which the governing equations are solved using the volume fraction in each cell.

The experimental equation was put to calculate the stable velocity in the literature [39] as follows:

(8) $$\begin{array}{c} V_{b}=kV^{m} \end{array}$$

(9) $$\begin{array}{c} k=\frac{25.0}{1.0+0.33Mo^{0.29}} \end{array}$$

(10) $$\begin{array}{c} m=0.167\left(1.0+0.34Mo^{0.24}\right) \end{array}$$

(11) $$\begin{array}{c} Mo=\frac{g{\mu_{\varepsilon }}^{4}△\rho }{{\rho_{c}}^{2}\sigma^{3}} \end{array}$$

V is the volume of the bubble, $m^{3}$; Mo is Morton number to describe the shape of the moving bubble, △ρ is the two–phase density subtraction, and the other physical properties are tannin-based foaming precursor resin.

### 2.2 Model validation

In this study, the rising behavior of a single bubble was simulated in still water to verify the rationality and reliability of the calculation model with experimental observations in the literature. The calculation domain of the single bubble rising condition is a 0.08 m width and 0.16 m height two–dimensional region, and the wall has no slip, the pressure outlet is set at the top, and the bottom is fixed. The initial radius of the bubble is 0.003 m, which is still symmetric in the calculation region far away from the bottom at 0.015 m, and the two-phase parameters are set in Table 1.

**Table 1:** Physical property parameters of the gas–liquid phase.

Phase	Density（kg∙m^−3^）	Viscosity（Pa∙s）	Surface tension（N∙m^−1^）
Air	1.225	1.7894e-05	–
Water	1000	0.001	0.07

**Fig. 1:**
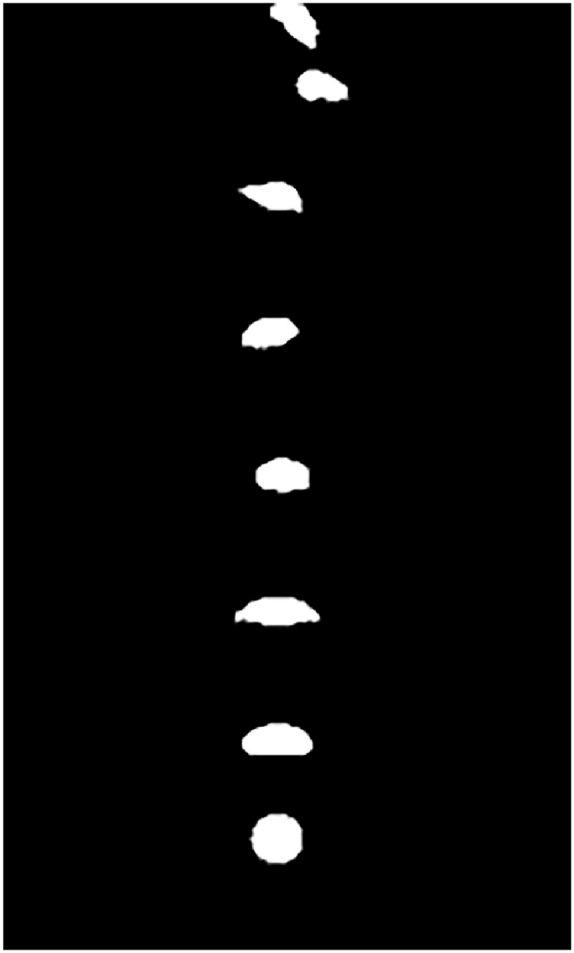
Floating process of a single bubble in water.

Fig. 1 shows the rising process of the air bubble in water for a basic analysis. It is clear to find that the bubble motion did not along with a vertical line raising path, but swings left and right. Additionally, the morphology feature of air bubbles has taken place remarkably. This phenomenon is caused by the resistance of liquid in the floating process which is similar to the literature reported [40].

**Fig. 2:**
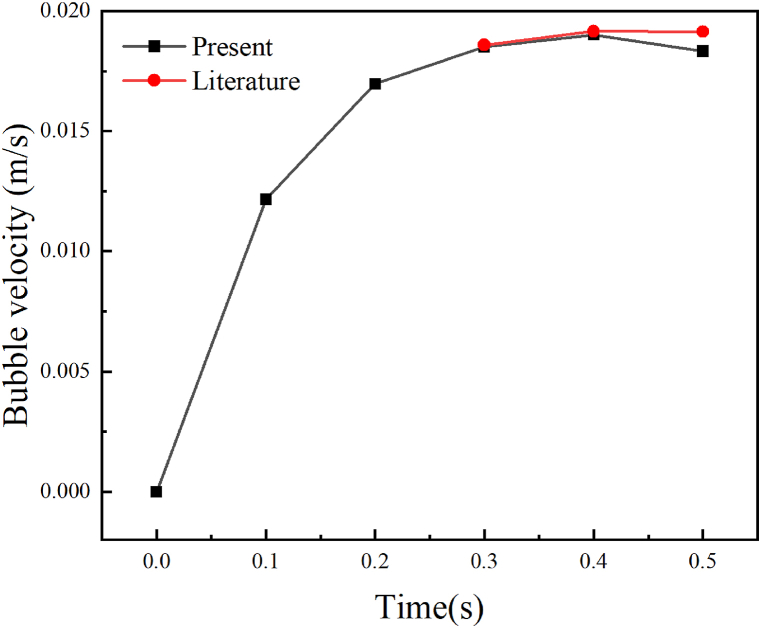
The bubble rising velocity in water.

Fig. 2 shows a theoretical calculation value on the velocity of air bubbles in water. The bubble movement experiment in water has been done and the results are reported in the literature [40]. Encouraged by the results, the theoretical calculation value shown in Fig. 2 is close to the practical experiment results, which confirms that the calculation model is reliable. This model will be utilized to simulate the air bubble motion behavior in tannin resin for visual observation of the results of experiment.

## 3 Results and discussion

### 3.1 Single bubble rising behavior in tannin-based foaming precursor resin

The rising behavior of the air bubble is deeply connected with the physical properties of tannin-based foaming precursor resin, especially in terms of viscosity and surface tension. Therefore, it is necessary to study the influence of changes in tannin-based foaming precursor resin viscosity and surface tension on the bubble rising behavior.

In the same calculation region as the water experiment in the validation part, the initial radius of the bubble is 0.005 m, and it is still symmetric to tannin-based foaming precursor resin, which is far away from the bottom by approximately 0.015 m. The physical properties of tannin-based foaming precursor resin and air are shown in Table 2. The physical properties were tested five times to get the average value. The density is determined by measuring the mass and volume of the tannin-based foaming precursor resin. The viscosity and surface tension were obtained directly using SNB-2 and BZY-1, respectively.

**Table 2:** Physical property parameters of the gas–liquid phase.

Phase	Density （kg∙m^−3^）	Viscosity （Pa∙s）	Surface tension（N∙m^−1^）
Air	1.225	1.7894e-05	–
Precursor	1421.667	0.4283	0.045

#### 3.1.1 Bubble rising behavior under various physical property conditions

**Fig. 3:**
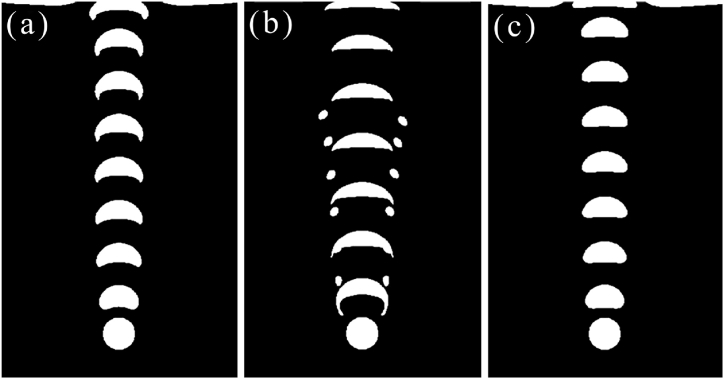
Floating process of a single bubble in tannin-based foaming precursor resin with variable properties. (a) Bubble rise history (μ = 0.4283, σ = 0.045), (b) Bubble rise history (μ = 0.04283, σ = 0.045), and (c) Bubble rise history (μ = 0.4283, σ = 0.1).

**Fig. 4:**
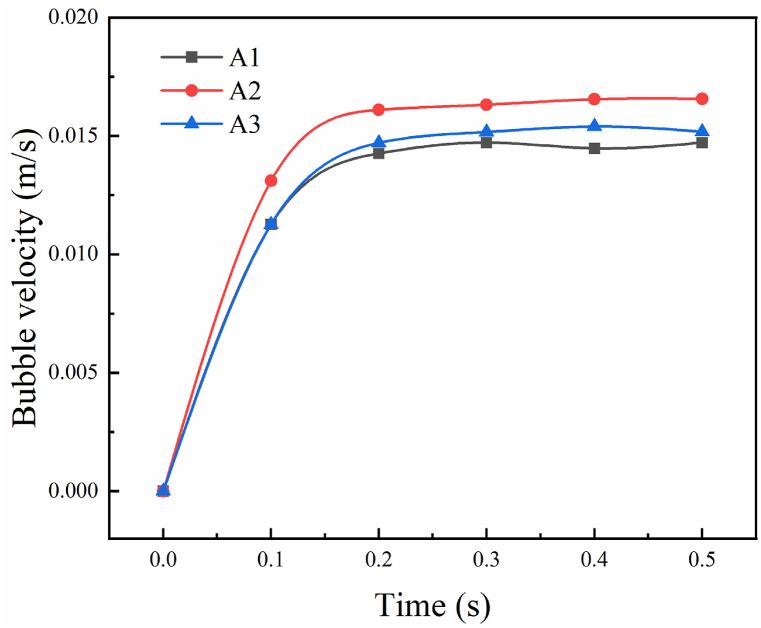
The bubble rising velocity in a variable tannin-based foaming precursor resin. (A1 is the velocity of condition in Fig. 3(a)–A2 is the velocity of condition in Fig. 3(b)–A3 is the velocity of condition in Fig. 3(c)).

The rising behavior of air bubbles in tannin-based resin was modeled by the calculation software based on the physical property parameters of the gas–liquid phase (as shown in Table 2), and the calculation results of bubble rising behavior are shown in Fig. 3 as the form of visual direct observation. For Fig. 3(a), the air bubble shape in the rising process gradually changes from spherical to hemispherical and finally becomes crescent while the viscosity of tannin resin foaming precursor is 0.4283 Pa∙s, and the corresponding surface tension is 0.045 N/m. The floating speed for air bubbles went down with the bubble shape turned to a crescent. It has a greater drag force as the greater top surface of air bubble formed with the shape change. This conclusion was proofed and shown in Fig. 4. In addition, with the large viscosity of tannin-based foaming precursor resin, the viscous resistance during the rise of the bubble is large, the rising speed is slow, yet the trajectory is not affected, keeping vertically rising. The stable velocity of the bubble is calculated by experimental equation (8) and the value is approximately v1 = 0.149578 m/s. This result is in good agreement with the simulation value, which proves that the model used to simulate the bubble behavior in tannin-based foaming precursor resin is reliable.

For discussing the floating behavior of air bubble was affected by the viscosity and surface tension separately, the dynamic viscosity was set to reduction by a factor of ten in this case, but keeping the same surface tension of 0.045 N/m. The decrease in viscosity accelerates the rising speed of the bubble, and the disturbance of the bubble to the tannin-based foaming precursor resin becomes larger. The bubble has a wake, and two symmetric vortices are produced around the bubble as shown in Fig. 3(b). With increasing of rising speed, the deformation of the bubble is large, similar to the cap shape.

When the viscosity is the same as that in case 3(a), the surface tension is 0.1 N/m, the deformation of the bubble is small but still changes from spherical to hemispherical, and the motion trajectory is basically not affected; it rises vertically as displayed in Fig. 3(c). It is easy to realize that the large surface tension resistance is smaller, the bubble rises faster, the bubble is more stable under various forces, and it is not easy to deform. The bubble's rising velocity according to a general trend, i.e., the final speed for the air bubble will increase gradually, to reach a stable value. The floating speed for air bubbles in tannin resin, identically, will trend to a constant value, as shown in Fig. 4.

Comparing the result of these conditions, bubbles rising in low viscosity caused greater deformation and were easier to escape, in the process of preparing tannin foam, while tannin-based foaming precursor resin has a greater viscosity which led to more bubbles staying in it, and finally the foam with lighter weight. At the same time, the shape of the bubble can also be controlled by adjusting the surface tension of the resin, which is beneficial to the construction of bubble micromorphology and then to acquiring functional foam.

#### 3.1.2 Bubble rising behavior under various bubble radius conditions

**Fig. 5:**
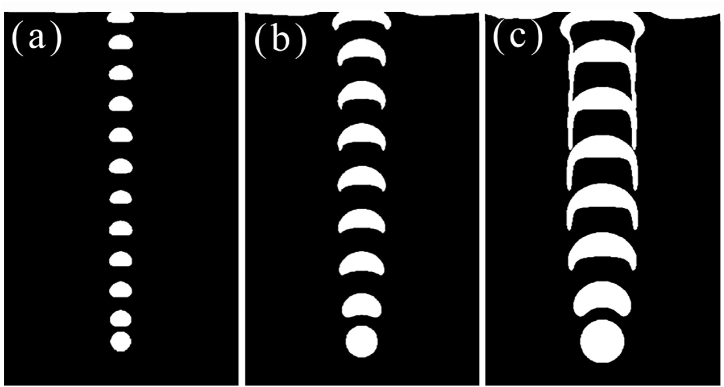
Floating process of variable bubble radius in tannin-based foaming precursor resin. (a) Bubble rise history (D = 0.003 m), (b) Bubble rise history (D = 0.005 m), (c) Bubble rise history (D = 0.007 m).

**Fig. 6:**
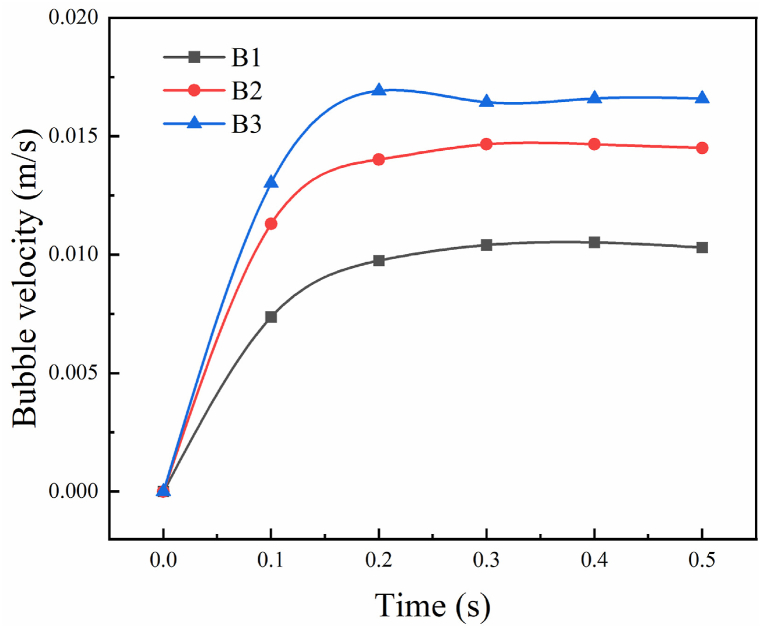
Bubble rising velocity with variable bubble radius. (B1 is the velocity of condition in Fig. 5(a), B2 is the velocity of condition in Fig. 5(b), B3 is the velocity of condition in Fig. 5(c)).

The bubble size and distribution are also important to the rising behavior. To investigate the deformation of the bubble radius, three bubble radii were simulated in tannin-based foaming precursor resin. The movement trajectory of the bubble is as follows:

When the bubble radius is 0.003 m as shown in Fig. 5(a), the bubble deformation is small, from spherical to hemispherical, and keeps rising vertically. When the bubble radius is 0.005 m as displayed in Fig. 5(b), the bubble deformation is more significant, from sphere to crescent and then gradually to cap shaped, and the movement trajectory remains straight up. When the radius of the bubble is 0.007 m like in Fig. 5(c), the bubble deformation is large, from spherical to crescent, and then quickly becomes cap shaped, fragmented bubbles appear at the tails of the two wings of the bubble, and the crescent part of the main body shrinks and rises vertically. As seen from the figure below, the larger the radius of the bubble, the more significant the deformation in the tannin-based foaming precursor resin, and the faster the rising speed as Fig. 6 shows. Therefore, to keep the shape of bubbles, getting smaller radius is beneficial for bubble growing.

### 3.2 Parallel double bubbles rising behavior in tannin-based foaming precursor resin

**Fig. 7:**
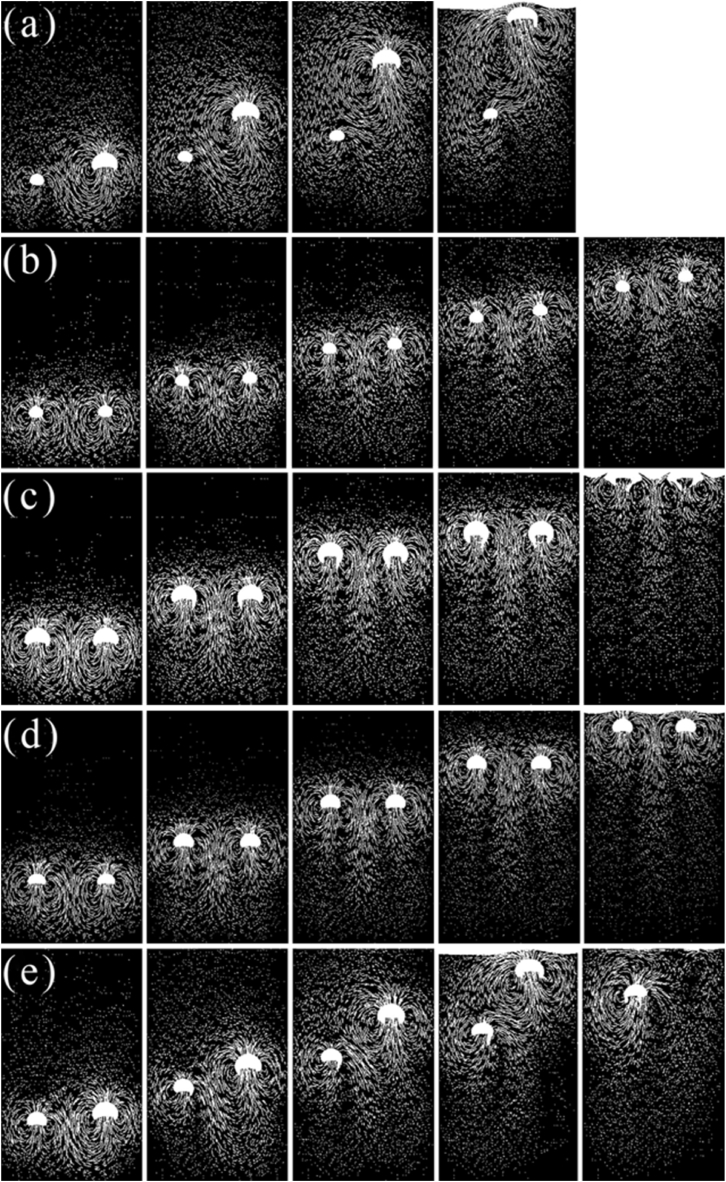
Study the rising behavior of double bubbles based on simulations. (a) Bubble shape and vector sequence (D1 = 0.003 m, D2 = 0.005 m), (b) Bubble shape and vector sequence (D1 = 0.003 m, D2 = 0.003 m), (c) Bubble shape and vector sequence (D1 = 0.005 m, D2 = 0.005 m), (d) Bubble shape and vector sequence (D1 = 0.004 m, D2 = 0.004 m) and (e) Bubble shape and vector sequence (D1 = 0.004 m, D2 = 0.005 m).

In the calculation region of 3.1, the rising process of parallel double bubbles is simulated and calculated, and the initial state of the bubbles is a horizontal symmetrical distribution. The calculation conditions are in Fig. 7.

Under condition 7(a), the degree of bubble deformation was significantly higher than that of condition 7(b). When the radius of the bubble was 0.005 m, the rising speed increased and then decreased, the 0.003 m bubble rising speed gradually decreased, and at the same time, the distance between the two bubbles gradually decreased, and the bubbles did not merge as shown in Fig. 7(a). The main reason for this phenomenon is that the wake area at the bottom of the bubble produces two vortex symmetrical structures in the bubble rising process, and the vortex structure continues to grow with the rise of the bubble. The rising speed of the large bubble is higher than the speed of the small bubble. When the bubble rises approximately 0.3 s, the small bubble will move in the wake area of the large bubble, affected by the large bubble vortex, the speed of the small bubble slows down, the pressure in the wake area is small, and the two bubbles gradually approach.

From the rising trajectory of the bubble in the 7(b), 7(c), and 7(d) working conditions, it is shown in Fig. 7(b) that the bubble deforms from a sphere to an ellipsoidal for a short time and then maintains the ellipsoidal, and in Fig. 7(d) bubbles in the process of rising from spherical to hemispherical and then gradually to crescent, keeping the crescent to rise until the bubble is stable, and the bubble deformation is significant. The vortex area is larger, and has the fastest rising speed, but the double bubble also does not merge and rising phenomena as displayed in Fig. 7(c) are the same as the literature [37]. The relative position of the two bubbles does not change significantly during the rising process, but the two bubbles have a slight tendency to move closer to the middle, which is due to the size, shape and initial position of the two bubbles being the same, resulting in the velocity vector of the wake area of the two bubbles being completely symmetrical, but due to the large viscosity of the tannin polymer solutions, the bubble deformation is not obvious, and the disturbance is reduced. The two bubbles appear near each other but still do not merge. The rising speed of double bubbles with a large radius is significantly greater than that of double bubbles with a small radius. When the flow time is 0.2 s, the bubbles reach a steady state.

Comparing the 7(a) with 7(e) conditions, it is obvious that the bubbles swing left and right as shown in Fig. 7(e), which is due to the smaller horizontal spacing between the two bubbles and the small difference in radius, resulting in the phenomenon of approach–separation–approach of the two bubbles during the rising process.

#### 3.2.1 Floating process of doubles with variable radii and positions

To investigate the influence of the distance between the parallel double bubbles for further study, the simulation was performed to calculate the two conditions, in which the bubble has one radius but a different distance and the other has a different radius but the same distance.

**Fig. 8:**
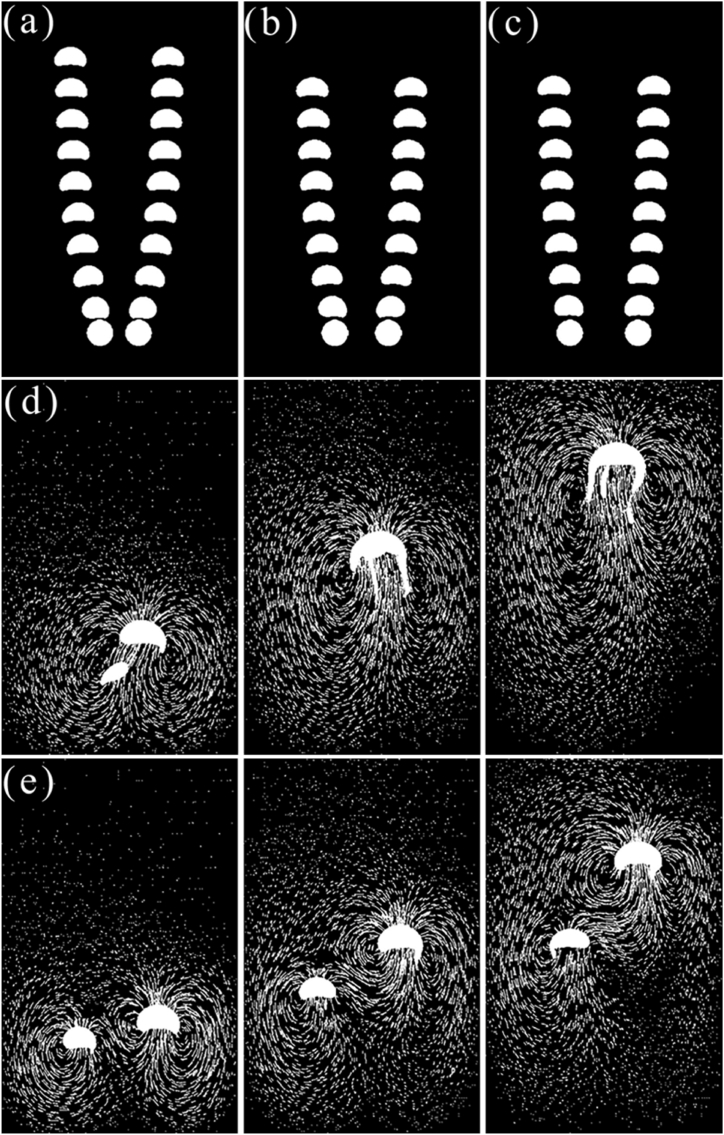
Floating process of bubbles with variable radii and positions.(a) Bubble rise history (D1 = 0.004 m, D2 = 0.004 m, L = 0.005 m), (b) Bubble rise history (D1 = 0.004 m, D2 = 0.004 m, L = 0.01 m), (c) Bubble rise history (D1 = 0.004 m, D2 = 0.004 m, L = 0.015 m), (d) Bubble shape sequence and vector (D1 = 0.003 m, D2 = 0.005 m, L = 0.005 m), (e) Bubble shape sequence and vector (D1 = 0.004 m, D2 = 0.005 m, L = 0.015 m).

Several distances in Fig. 8 are selected from a large number of pre-experiments to analyze the motion behavior of the double bubbles. The calculation result in Fig. 8(a) shows that the change in the distance in double bubbles is not significant in the same bubble radius. Under conditions 8(a), 8(b), and 8(c), the shape of the bubble changes from spherical to hemispherical. Compared to condition 8(d), the double bubbles are far away from each other, so when the bubble distance is less than 0.015 m, the trajectory of the bubble shows the trend of outside like in Fig. 8(a)–. (b) and (c), when the distance is more than 0.040 m, the trajectory remains straight up as shown in Fig. 7(d).

In the case of condition 8(d), the double bubble presents fusion, which can be easily observed in Fig. 8(d), when the distance is closer, and the bubble is bigger, the initial rising speed is larger, which was verified in section 3.1, so the flow time at 0.1 s, the small bubble is rising in the vortex of the large bubble. Due to the influence of the large bubble, the shape of the bubble changes from spherical to ellipsoidal, and at the same time, the rising speed of the small bubble is faster and finally it fuses with the large bubble and the volume is bigger, while the speed is faster. In the case of 8(e) conditions, the double bubble does not fuse as displayed in Fig. 8(e). Compared to the 8(e) condition in 3.2, due to the near distance, the disturbance is significant, the small bubble is in the vortex of the large bubble, and the rising speed is increased. Under both conditions, the small bubble shows different motion trends. The reason is that when the two bubbles are far away from each other, the large bubble has a faster speed, and then the small bubble does not enter the disturbance region right now, which is generated by the large bubble but still affected by the force, resulting in the side near the large bubble of the small bubble having a lower speed. When the distance between the two bubbles is nearer, the small bubble is in the vortex of the large bubble, with the change in pressure, resulting in the side near the large bubble of the small bubble having a larger speed.

### 3.3 Coaxial double bubbles rising behavior in tannin-based foaming precursor resin

In the environment of 3.1, the rising process of coaxial double bubbles was calculated, and the initial state of the bubble is still symmetric to the tannin-based foaming precursor resin.

**Fig. 9:**
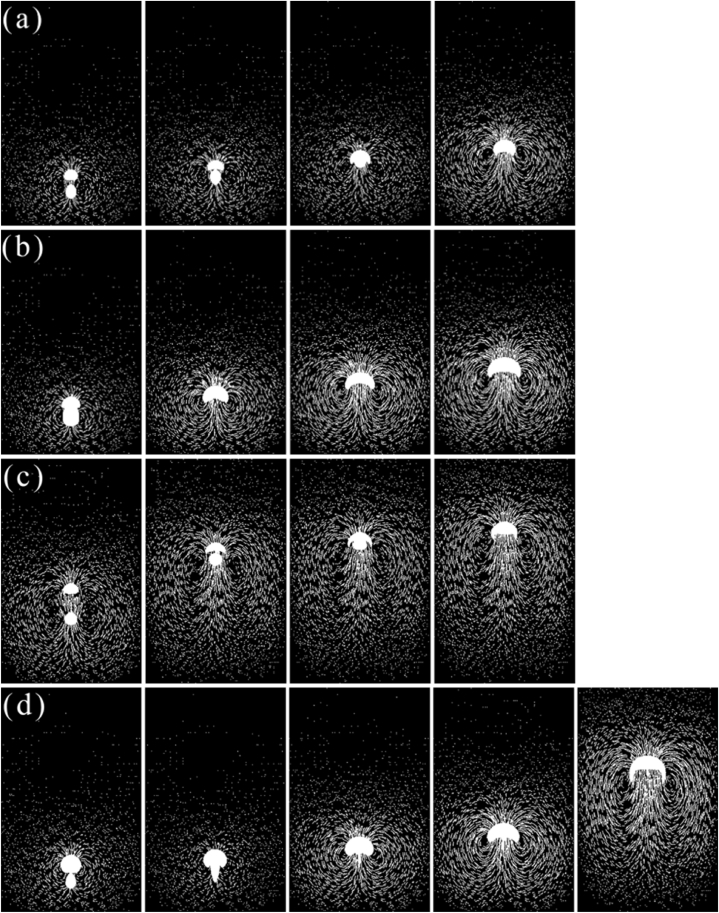
Floating process of coaxial double bubbles in tannin-based foaming precursor resin. (a) Bubble coalescence vector (D1 = 0.003 m, D2 = 0.003 m, L = 0.01 m), (b) Bubble coalescence vector (D1 = 0.003 m, D2 = 0.005 m, L = 0.01 m), (c) Bubble coalescence vector (D1 = 0.003 m, D2 = 0.003 m, L = 0.02 m), (d) Bubble coalescence vector (D1 = 0.005 m, D2 = 0.003 m, L = 0.01 m).

As shown in Fig. 9, in the case of 9(a), 9(b), 9(c) and 9(d) conditions, the double bubble presents fusion, and the figure below is the vector diagram of double bubbles at 0.2 s. Comparing the conditions of 9(a) and 9(c), when the distance in the coaxial double bubble is large, the speed is lower for the bubbles to fuse and at the same time the upper bubble has a significant deformation as shown in Fig. 9(a) and (c). Comparing the conditions of 9(a), 9(b) and 9(d), when the radius of the bubble is large, the fusion speed is faster like in Fig. 9(b), and when the radius of the upper bubble is smaller than the other, the fusion time is shorter and the sum of volume is smaller as displayed in Fig. 9(d). Hence the growing speed is affected by the radius and relative positions of bubbles.

## 4 Conclusions

The gas-liquid two-phase flow in tannin-based foaming precursor resin was simulated under several conditions. The results show that by adjusting the surface tension and viscosity of the tannin-based foaming precursor resin, bubbles can be prevented from escaping and deforming, so as to obtain high porosity foam or other foam characteristics. The motion process of parallel bubbles is symmetric, and their motion properties depend not only on the properties of the solution, but also on the relative position of the bubbles, which is crucial for bubble merger. When the bubble distance is less than 0.015m, the bubble trajectory shows an outward trend, and when the bubble distance is greater than 0.040m, the trajectory basically maintains a straight line upward. However, due to the large number of bubbles, it is difficult to adjust the distance when actually preparing the foam. Therefore, it is necessary to understand the movement of bubbles in tannin-based foaming precursor resin for subsequent bubble fusion and rupture analysis. For coaxial double bubbles, the results show that there is a difference in the growth rate of bubbles during the foaming process, which will lead to the formation of foam defects. Therefore, understanding the growth process of bubbles is an indispensable part of trying to regulate the micropore structure of tannin foams, which will provide theoretical guidance for obtaining good tannin foams. In fact, the actual foaming process of tannin foam materials involves the change of energy, and the energy change in the foaming process needs to be added for analysis and calculation in future research, to improve the simulation of the foaming process of tannin foam materials under chemical reaction.

## CRediT authorship contribution statement

**Lan Huang:** Writing – original draft, Methodology, Conceptualization, Writing – original draft, Methodology, Conceptualization. **Haizhu Wu:** Supervision. **Wenbin Yuan:** Supervision. **Hisham Essawy:** Writing – review & editing, Supervision. **Guanben Du:** Supervision, Funding acquisition. **Xiaojian Zhou:** Supervision, Funding acquisition. **Xinyi Chen:** Writing – review & editing, Supervision, Project administration, Funding acquisition.

## Data availability statement

All data generated or analyzed during this study are included in this published article.

## Declaration of competing interest

The authors declare that they have no known competing financial interests or personal relationships that could have appeared to influence the work reported in this paper.
